# Running in the heat reduces time to task failure, attenuates biomarkers of muscle microtrauma, and exacerbates low-frequency force depression and cardiac vagal withdrawal over 72-h post-exercise

**DOI:** 10.1007/s00421-026-06367-7

**Published:** 2026-07-25

**Authors:** Yago Medeiros Dutra, Paloma Tavares Mendonça, João Pedro Fialho Lopes, Michael R. Esco, Elvis de Souza Malta, Rodrigo Araújo Bonetti de Poli, Alessandro Moura Zagatto

**Affiliations:** 1 Laboratory of Physiology and Sport Performance (LAFIDE), School of Sciences, São Paulo State University (UNESP), Bauru, Brazil; 2 Exercise Physiology Laboratory, Department of Kinesiology, University of Alabama, Tuscaloosa, AL USA; 3 College of Health and Life Sciences, Hamad Bin Khalifa University, Doha, Qatar; 4 Department of Physical Education, School of Sciences, Sao Paulo State University (UNESP), Av. Eng. Luiz Edmundo Carrijo Coube, 14-01, Vargem Limpa, Bauru, CEP 17033- 360 SP Brazil

**Keywords:** Running performance, Heat, Twitch interpolation, HRV, Recovery

## Abstract

**Purpose:**

As exercise in the heat becomes increasingly common, a better understanding of post-exercise responses is essential to guide the appropriate use of fatigability monitoring tools and recovery strategies. Therefore, this study investigated the effect of running in the heat on neuromuscular function, cardiac autonomic modulation, and plasma markers of muscle microtrauma during post-exercise recovery.

**Methods:**

Twelve participants (V̇O_2max_: 44.8 ± 3.5 mL/kg/min) ran to task failure at a velocity associated with the respiratory compensation point (vRCP) in temperate (22.4 ± 0.9 °C) and hot (38.8 ± 0.7 °C) environments. Voluntary and evoked contractions of the knee-extensors, heart rate variability (HRV), and plasma creatine kinase (CK) and lactate dehydrogenase (LDH) concentrations were assessed pre-exercise, within the first hour post-exercise, and at 24-, 48, and 72-h post-exercise.

**Results:**

Running in the heat reduced time to task failure (TTF, 17.8 ± 4.4 min vs. 42.3 ± 20.1, *p* < 0.01, *d* = 1.96) and attenuated the increase in plasma CK at 24 h post-exercise (condition×time interaction, post-hoc *p* < 0.05). Neuromuscular function and cardiac autonomic modulation were impaired within the first hour post-exercise (time effects, *p* < 0.05) and returned to baseline values at 24-h post-exercise in both conditions (post-hoc *p* > 0.05). Aggregated data (condition effects, post-hoc *p* < 0.05) over the 72-h monitoring time evidenced exacerbation of low-frequency force depression (reduced Db10_Force_/Db100_Force_) and cardiac vagal withdrawal (reduced high-frequency HRV power) after running in the heat.

**Conclusion:**

Running in the heat at vRCP reduced TTF. Although heat reduced exercise duration, it specifically exacerbated low-frequency force depression and cardiac vagal withdrawal for 72-h post-exercise while attenuating the increase in plasma markers of muscle microtrauma at 24-h post-exercise.

**Supplementary Information:**

The online version contains supplementary material available at https://doi.org/10.1007/s00421-026-06367-7.

## Introduction

Major sporting events (e.g., FIFA World Cup and Summer Olympic Games) have increasingly been held in hot environments (Carmo et al. 2026; Craig and Karabas 2024), and climate projections indicate that heat waves will become more frequent and severe in the coming years (Perkins-Kirkpatrick and Lewis 2020). As such, heat presents an inherent challenge for professional, amateur, and recreational athletes who must maintain their habitual exercise demands (e.g., time spent in a target and planned exercise intensity) during training and competition (Craig and Karabas 2024). Exercising in the heat induces profound homeostatic perturbations, including increased cardiovascular strain, accelerated muscle glycogenolysis, and sarcoplasmic reticulum dysfunction (Périard et al. 2021; Roberts et al. 2023). While these perturbations are well established as contributors to impaired performance in the heat, particularly during endurance tasks (de Korte et al. 2021a; Galloway and Maughan 1997; Mitchell et al. 2014; Nielsen et al. 1993), their influence on post-exercise disturbances and recovery remains poorly understood (Périard et al. 2021).

For example, Nybo et al. (2013) reported similar levels of knee-extensors peak force, contractile function, and voluntary activation 24 to 48 h after soccer matches played at 43 °C and 21 °C. However, whether neuromuscular function is affected within minutes to hours after exercising in the heat remains insufficiently explored. Rodrigues-Júnior et al. (2023) observed that the reduction in countermovement jump height one hour following a 10-km time-trial was not influenced by heat stress (i.e., exercising at 25 °C vs. 35 °C). Nevertheless, the countermovement jump presents inherent limitations for assessing neuromuscular function (Gathercole et al. 2014), thereby precluding definitive conclusions regarding the short-term effects of exercise in a hot environment on post-exercise neuromuscular function.

Importantly, post-exercise fatigability is characterized not only by reduced force output but also by broader homeostatic perturbations, including disturbances in cardiac vagal modulation (Behrens et al. 2023; Enoka and Duchateau 2016). Given that both heat stress and exercise independently reduce cardiac vagal modulation (Brenner et al. 1997), monitoring autonomic responses may provide a more comprehensive understanding of the physiological disturbances induced by exercise in the heat. In addition, exercising in the heat has also been suggested to exacerbate exercise-induced disruption of muscle integrity, particularly in untrained individuals (Roberts et al. 2023). Accordingly, assessing plasma biomarkers of muscle microtrauma, such as creatine kinase (CK) and lactate dehydrogenase (LDH), may offer further insight into the homeostatic disturbances associated with exercise in this environment (Halson 2014).

A more detailed understanding of fatigability and physiological disturbances following exercising in the heat may inform the selection of appropriate monitoring tools and recovery strategies tailored to this specific condition, thereby mitigating performance decrements and reducing potential health risks (Kellmann et al. 2018). Therefore, the aim of the present study was to characterize post-exercise responses following a fixed-workload run performed in the heat and to determine whether the magnitude and time course of these responses differ from those observed after exercise in a temperate environment. A comprehensive assessment of post-exercise disturbances was conducted at multiple time points (immediately, 15-, 30-, 45-, and 60-min, as well as 24-, 48-, and 72-h), evaluating global, central, and peripheral aspects of neuromuscular function, cardiac autonomic modulation, and plasma markers of muscle microtrauma. We hypothesized that exercising in the heat would impair performance and amplify disturbances in neuromuscular function, cardiac autonomic modulation, and muscle integrity, resulting in a delayed recovery to baseline values.

## Methods

### Participants

A minimum of ten participants was estimated to achieve a statistical power of 0.90 in *F* tests, with α set at 0.05. This estimation was based on Goodall et al. (2015), who observed decreases in peak voluntary force of knee extensors of 13 ± 9% and 9 ± 5% after matched-duration cycling in the heat (34 °C) and temperate (18 °C) conditions, respectively. The effect size of this difference (Cohen’s *d* = 0.57) was converted to an effect size *F* of 0.28 using a previously suggested equation (Cohen 2013). The G*power 3.1 software (Faul et al. 2007) was used to power analysis and the input parameters for the software were *ANOVA: repeated measures*,* within factors*; *Effect size f: 0.28; Number of groups: 2; Number of measurements: 6; Correlation among repeated measures: 0.8* (a ballpark value for neuromuscular function outcomes according to data from our research group (Dutra et al. 2023); *nonsphericity correction: 1; Options: as in Gpower 3.0.* To account for potential dropouts, we recruited 15 healthy, recreationally active individuals. Eligibility criteria included no medical or nutritional treatment in the preceding 6 months and the absence of neurological, cardiovascular, or musculoskeletal disorders that could affect the study outcomes. All participants provided written informed consent after being fully informed of the experimental procedures. Due to successive absence from the trials (*n* = 3), the final sample consisted of 12 participants (10 males, 2 females; age = 24 ± 4 years old; stature = 171.1 ± 6.9 cm; body mass = 75.0 ± 12.6 kg; training volume = 11.5 ± 6.3 km/week). The two female participants reported no menstrual cycle-related irregularities (e.g., amenorrhea), gynaecological conditions (e.g., endometriosis), or pregnancy. They reported use of a combined monophasic oral contraceptive pill and a menstrual cycle length of 28 ± 3 days for at least 6 months prior to participation. For both female participants, the experimental trials conducted in temperate and hot environmental conditions were completed within 8 days following the end of menstruation. The study was approved by the Sao Paulo State University Research Committee (Number 5.717.323) and conducted in accordance with the Declaration of Helsinki.

### Experimental design

**Fig. 1:**
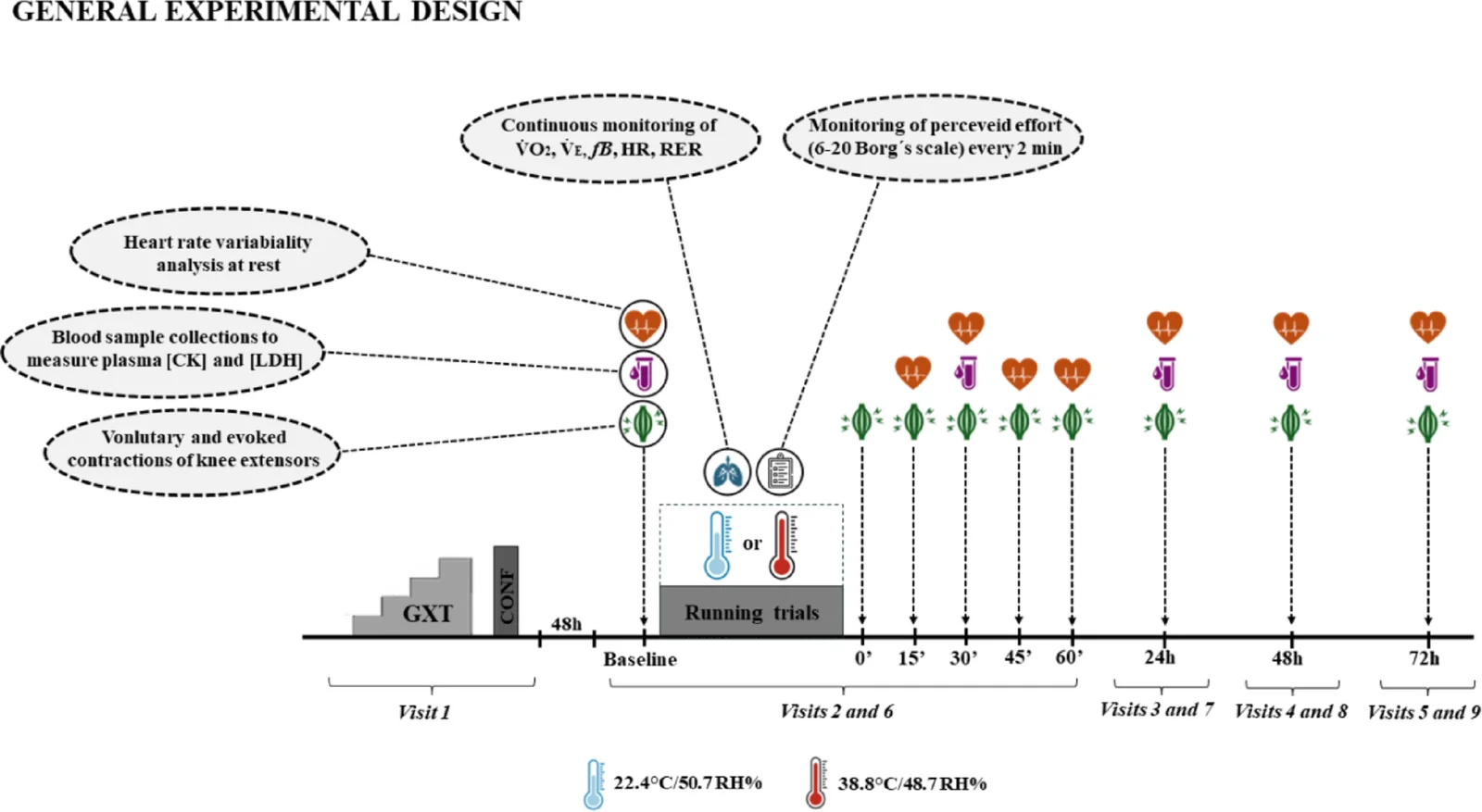
Schematic representation of the investigation design. *CWR:* Constant work-rate; *GXT:* Graded exercise test; *vRCP:* Velocity associated to respiratory compensation point; *CK:* Creatine kinase concentration; *LDH:* Lactate dehydrogenase concentration; *V̇O*_*2*_:Rate of oxygen consumption; *V̇O*_*2*_max: Maximal rate of oxygen consumption; *V̇E*: Ventilation; *PetCO*_*2*_: Partial expiratory pressure of carbon dioxide; *HR*: Heart rate; *RER*: Respiratory exchange ratio

The experimental design is illustrated in Fig. 1. Participants attended the laboratory on nine occasions and were instructed to abstain from food for 2 h, caffeinated beverages for 8 h, and moderate-to-vigorous physical activity for 24 h prior to each visit. During the first visit, stature and body mass were measured, followed by a graded exercise test (GXT) and familiarization with study procedures (i.e., peripheral electrical stimulation, maximal knee-extensor contractions, and rating scales). During subsequent visits, participants performed constant work-rate (CWR) runs at a velocity that corresponded to the respiratory compensation point (vRCP) until failure in both a hot (38.8 ± 0.7 °C; 48.7 ± 2.1% relative humidity) and temperate/control environment (22.4 ± 0.9 °C; 50.7 ± 1.5% relative humidity). These exercise trials were performed one week apart in a randomized order. Seven participants (*n* = 7) performed the CWR run in the heat during the second visit (i.e., first exercise trial).

During running trials, cardiorespiratory responses were continuously recorded, and ratings of perceived exertion (RPE) were assessed every 2 min using the Borg 6–20 scale (Borg 1982). HRV, plasma biomarkers of muscle microtrauma (CK and LDH), and neuromuscular function were assessed at baseline and at multiple recovery time points, except immediately post-exercise when only neuromuscular function was assessed. Specific measurement time points for each variable are described in detail in subsequent sections. All procedures were conducted in a temperate environment (22.4 ± 0.9 °C), except for neuromuscular assessments immediately post-exercise in the hot condition, which were performed at the same temperature as the running trial. The study was conducted during the Southern Hemisphere autumn, a period when local mean temperatures typically range from 18 to 26 °C.

### Exercise protocols

All running efforts (i.e., GXT and CWR tests) were performed on a motorized treadmill (ATL, Inbramed, Brazil). Participants wore a safety belt attached to an overhead support to ensure maximal effort and minimize risks of falls and injuries. Task failure was determined as the point at which participants voluntarily disengaged from exercise despite strong verbal encouragement.

### Graded exercise test

The GXT was performed to determine the respiratory compensation point (RCP), maximal oxygen uptake (V̇O_2max_), and their corresponding velocities (vRCP and velocity at V̇O_2max_ [vV̇O_2max_], respectively). The test began at 8 km/h for males and 6.5 km/h for females, with velocity increased by 1.5 km/h every 2 min until task failure (Bentley et al. 2007). The starting step for females was adjusted based on previous findings of our research group (de Poli et al. 2021; Minuzzi et al. 2022) to ensure the accomplishment of previous recommendations for GXT tests (e.g., start at 60 to 70% of the participants’ V̇O_2max_, leading to task failure in about 8 to 12 min) (Bentley et al. 2007). The treadmill gradient was fixed at 1% throughout the test (Bentley et al. 2007). Five minutes after the end of the GXT, participants completed a constant-load run at a velocity 5% higher than the peak velocity achieved during the test. This effort was performed to add confidence in the establishment of a V̇O_2_ response more likely to reflect the true V̇O_2max_ (Keir et al. 2014).

### CWR tests and environmental conditions

The CWR tests were performed at vRCP, starting 2 min after a standardized warm-up (5 min at 8 km/h for males and at 6.5 km/h for females)(Dutra et al. 2026). Running was chosen because it is a key component of training in many individual and team sports (Buchheit and Laursen 2013). The vRCP was selected because it relates to the boundary between heavy- and severe-intensity domains, eliciting pronounced metabolic perturbation while permitting a tolerable exercise duration (Behrens et al. 2023). The same exercise intensity (i.e., vRCP) was used in both conditions to investigate the impact of heat on performance and post-exercise responses at a consistent work rate. This approach likely mirrors the fixed external demands, such as specific running velocity, that athletes typically strive to maintain during training and competition, regardless of environmental conditions (de Korte et al. 2021b). Warm-ups were conducted under the same environmental conditions as the corresponding trial. The time to task failure was expressed in minutes and recorded as a performance marker. Participants were not informed of their time to task failure performance from either trial until all study procedures had been completed.

The CWR trials were conducted in a 7.5 m² room enclosed by plastic walls to isolate heat and maintain stable thermal conditions. In the hot condition, four electric heaters (model AB1100N, Britânia, Brazil) were positioned in the corners of the room and one in front of the treadmill, which ensured that the temperature was maintained between 38 and 40 °C. If the temperature fell below or increased above this range, the heaters were reactivated or deactivated, respectively. In the temperate condition, an air conditioner with a thermostat (Elgin, Brazil) regulated the temperature at 22 °C (± 1 °C). Room temperature was monitored every 5 min using a centrally located mercury thermometer.

### Data collection and analysis

#### Respiratory data

Breath-by-breath pulmonary gas exchange and ventilation (V̇_E_) were continuously measured using a metabolic cart (Quark, CPET, COSMED, Italy) coupled with a chest strap (Polar H10, Polar Electro Oy, Kempele, Finland). In accordance with the manufacturer’s recommendations, the metabolic cart was calibrated with gas of known concentration (16% O_2_ and 5% CO_2_) prior to all tests. Aberrant breaths were defined as values ± 3 SD from the local mean and were excluded (Keir et al. 2014). Breath-by-breath data were then interpolated every 1 s (Keir et al. 2014) using OriginPro v8.0 software (Origin Lab Corporation, United States).

V̇O_2max_ was determined as the highest 20-s average of V̇O_2_ during the final stage of the GXT. However, if a value more than 2.1 mL/kg/min higher was observed during the subsequent constant-load run, that value was recorded as V̇O_2max_ (Keir et al. 2014). The minimal velocity at which V̇O_2max_ was achieved was recorded as vV̇O_2max_ (Keir et al. 2014).

For the estimation of RCP, raw value profiles of V̇_E_, carbon dioxide output (V̇CO_2_), their ratio (V̇_E_/V̇CO_2_), and end-tidal pressure of CO_2_ (PetCO_2_) were plotted against time. RCP was identified as the point at which PetCO₂ began a sharp decline following a period of isocapnia, concomitant with the first breakpoint in V̇E/V̇CO₂ and the second breakpoint in V̇E (Keir et al. 2022). The running velocity at the GXT stage in which RCP occurred was recorded as vRCP. When necessary, vV̇O_2max_ and vRCP were adjusted according to Kuipers et al. (1985).

The test protocols used in this study did not account for the mean response time of respiratory V̇O_2_ or the non-linear relationship between work-rate and V̇O_2_ above lactate threshold (Keir et al. 2014). Therefore, the vRCP reported may reflect a work rate exceeding the maximum metabolic steady state. Consequently, the findings may not fully align with responses typically observed during high-intensity exercise.

During the CWRs, V̇O_2_, heart rate (HR), V̇_E_, respiratory exchange ratio (RER), and breathing frequency (*f*B) were measured for the following time points: initial 30 s, each 2 min of exercise until the shortest time to task failure observed among participants in both conditions, and at task failure. The data were calculated as the average of the last 20 s of each window and were expressed in raw values (Dutra et al. 2020).

#### Neuromuscular function

Across both CWR trials, neuromuscular assessments were performed at baseline, immediately post-exercise (within ~ 1-min), at 15-, 30-, and 60-min, as well as at 24-, 48-, and 72-h post-exercise. For each assessment, participants were seated in a custom chair with hips and knees fixed at 90° flexion. While in this position, they performed 5-s isometric maximal voluntary contractions (iMVCs) of the dominant knee-extensors, with peripheral nerve stimulation (PNS) applied during force production and at rest. Muscular force was acquired using a load cell (MK Controle, Brazil) attached to the ankle immediately above the medial malleolus by a metal rod secured with heavy-duty Velcro straps. During iMVCs, the surface electromyography activity (EMG) of the vastus lateralis was also recorded using self-adhesive Ag/AgCl surface electrodes (Meditrace 100, USA). The active electrode pair was positioned over the distal one-third of the muscle, and the ground electrode was placed on the patella. Signals were amplified (FE136, ADInstruments, New Zealand) and the EMG data, along with muscular force, were recorded using LabChart 8 software (ADInstruments, New Zeland) (Dutra et al. 2023).

Femoral nerve stimulations (pulse duration: 1 ms; square wave) were delivered using a high-voltage electrical stimulator (Bioestimulador V2 400 V peak-to-peak; Insight, Brazil). Stimulations were delivered through Ag/AgCl electrodes (10-mm of diameter, Kendall Medi-Trace, USA) placed over the femoral triangle (cathode) and carbon rubber electrodes (32.5 mm in diameter, CARCI, Brazil) placed over the greater trochanter (anode).

To assess voluntary activation (VA_PNS_), doublet high-frequency stimulations (100 Hz) were delivered at the visual peak force of the iMVC effort (~ 3-s after contraction onset) and again at 3-s after iMVC. Contractile properties were evaluated using single and doublet low-frequency (10 Hz) stimulations delivered to the relaxed muscle at 7- and 10-s after iMVC, respectively. Stimulus intensity was individually determined in each visit by applying single twitches to the relaxed muscle, starting at 30 mA, and increasing in 30 mA increments until a force plateau was (287 ± 112 mA across the trial). The final PNS intensity was set at 30% above this threshold to ensure full activation (Dutra et al. 2023).

All assessments were performed 2-min after two preliminary iMVCs (3 s each), separated by a 30-s rest interval, except for those conducted immediately post-exercise. These iMVCs served as warm-up, and the peak force achieved was recorded to confirm that participants reached maximal force during neuromuscular assessment (Button and Behm 2008).

The highest voluntary force recorded within a 100 ms period during the plateau of the iMVC was defined as the peak voluntary force (iMVC_Force_) and used as a global marker of neuromuscular function. To assess VA_PNS_, the superimposed force of the doublet evoked during the iMVC, and the force evoked by the potentiated doublet at rest after iMVC (Db100_Force_) were analyzed. The VA_PNS_ was calculated with the following equation (Strojnik and Komi 1998):

$$\:\mathrm{VA}\mathrm{PNS}\text{}\left(\mathrm{\%}\right)\mathrm{=}\left[\mathrm{1-\:}\frac{\mathrm{superimposed\:force\:}\left(\frac{\mathrm{force\:level\:at\:stimulation}}{{\mathrm{iMVC}}_{\mathrm{Force}}}\right)}{{\mathrm{Db100}}_{\mathrm{Force}}}\right]\mathrm{\:x\:100}$$

The potentiated peak force from the single stimulus (Qtw_pot_) was used as a marker of contractile function. Low-frequency force depression was assessed as the ratio between the force evoked by the doublet low-frequency stimuli (Db10_Force_) and Db100_Force_ (i.e., Db10_Force_/Db100_Force_ ratio). For EMG analysis, the signal was band-pass filtered (20–500 Hz). The amplitude of EMG response evoked by the single stimulus (M-wave amplitude) was recorded as an indicator of sarcolemmal excitability. The root mean square of the *vastus lateralis* EMG signal (RMS_VL_) was determined over a 0.5 s period around iMVC_Force_. The RMS/M-wave_amp_ ratio was calculated to estimate the neural drive to *vastus lateralis* (i.e., propagation of electrical signals from the central nervous system to the muscle) (Millet et al. 2011). All analyses were performed using the LabChart 8 software (ADInstruments, New Zealand). Data were expressed as a percentage change from baseline. The complete raw data for the knee-extensors function outcomes evaluated are available in the Supplementary Information (Document_1_SuppInfo.doc).

#### Heart rate variability

HRV was recorded at baseline, 15-, 30-, and 60-min, and at 24-, 48-, and 72-h post-exercise for each CWR running trial. At each time point, heart rate and rhythm were assessed via electrocardiography for a 6-min period while participants remained seated in a dimly lit, quiet room. Breathing frequency was not controlled. Surface Ag-AgCl electrodes (Meditrace 100, USA) were positioned in a modified lead-II configuration and connected to a bio-amplifier (FE136, ADInstruments, New Zealand) and a PowerLab system (16/35, ADInstruments, New Zealand) (Esco et al. 2017). Electrode sites were cleaned with rubbing alcohol prior to placement.

The final 5-minute segment of each electrocardiography recording was used for offline HRV analysis using LabChart 8 software (AD Instruments, New Zealand). The following indices were calculated: root-mean-square of successive differences of R-R intervals (RMSSD), the standard deviation of N-N intervals (SDNN), low-frequency (LF) power, high-frequency (HF) power, the standard deviation perpendicular to the line of identity (SD1), and the standard deviation parallel to the line of identity (SD2). Each HRV metric was log-transformed (Log10) to improve normality (Dutra et al. 2025; Esco et al. 2017) and expressed as a percentage change from baseline. The complete raw data for the HRV metrics evaluated are available in the Supplementary Information (Document_1_SuppInfo.doc).

#### Blood samples

Plasma concentrations of CK and LDH were analyzed as markers of muscle microtrauma (Brancaccio et al. 2010). Blood samples were collected at baseline and at 30-min, 24-, 48-, and 72-h after each CWR trial. Approximately 3 mL of venous blood was collected from the antecubital vein at rest, immediately centrifuged at 3,000 rpm for 15 min at 4 °C, and the extracted plasma was stored at − 80 °C for subsequent analysis. CK and LDH concentrations were determined using specific assay kits (LaborLab, Brazil; CK-NAC 1770070 and LDH-P UV 1770210) according to the manufacturer’s instructions. Data acquisition and analysis were performed using a spectrophotometer cuvette system coupled with Epoch 2 and Gen5 software (Biotek, USA). Analyses were made in duplicate, and the mean coefficient of variation was 6.4% for CK and 10.6% for LDH. Data were expressed as a percentage change from baseline. The complete raw data for the CK and LDH concentrations are available in the Supplementary Information (Document_1_SuppInfo.doc).

### Statistical analysis

All data are presented as mean ± standard deviation. Statistical significance was set at α of 0.05. Normality of data was verified using the Shapiro-Wilk test. A paired *t-*test was used to compare the time to task failure between conditions. The Cohen´s *d* was calculated to estimate effect size, with values of 0.2, 0.5, and, 0.8 representing small, moderate, and large effects, respectively (Lakens 2013). A two-way repeated measures ANOVA (condition × time) was used to compare the remaining outcomes between conditions over time. Assumptions of sphericity were explored and controlled for all variables with the Greenhouse–Geisser adjustment, where necessary. If significant main effects or interactions (condition × time) were observed, a Bonferroni post-hoc pairwise comparison was performed. Partial eta squared ($\:{\eta}_{\mathrm{p}}^{\mathrm{2}}$) was calculated to estimate effect size with values of 0.01, 0.06, and 0.14 representing small, moderate, and large effects, respectively (Hopkins et al. 2009). Within-condition changes from baseline were calculated as expressed as percentage. Between-condition differences were calculated as the change from baseline in heat condition *minus* that in temperate condition, also expressed as a percentage. Pearson’s correlation coefficients were used to assess the relationship between the studied variables’ percentage differences. The magnitude of the correlation was interpreted using thresholds of 0 to 0.1 (trivial), 0.11 to 0.3 (small), 0.31 to 0.5 (moderate), 0.51 to 0.7 (large), 0.71 to 0.9 (very large) (Hopkins et al. 2009). The SPSS software package version 20.0 (IBM Corp., USA) was used for all statistical analysis.

## Results

Maximal and submaximal responses recorded during GXT are displayed in Table 1.

**Table 1:** Maximal and submaximal physiological parameters measured during GXT

Parameter	Mean	±	SD	(range)
V̇O_2max_ (L/min)	3.3	±	0.4	(2.64 to 3.78)
V̇O_2max_ (mL/kg/min)	44.8	±	3.5	(39.0 to 48.5)
vV̇O_2max_ (km/h)	14.4	±	1.5	(12.8 to 16.4)
Estimated RCP				
V̇O_2_ (L/min)	2.8	±	0.3	(2.2 to 3.3)
Velocity (km/h)	10.1	±	1.2	(9.0 to 12.8)

Data are presented as mean ± standard deviation (95% of confidence interval). *V̇O*_*2max*_: Maximal oxygen consumption; *vV̇O*_*2max*_: Velocity associated to V̇O_*2max*_; *RCP*: Respiratory compensation point; *V̇O*_*2*_: Oxygen consumption

### Time to task failure and exercise responses

**Fig. 2:**
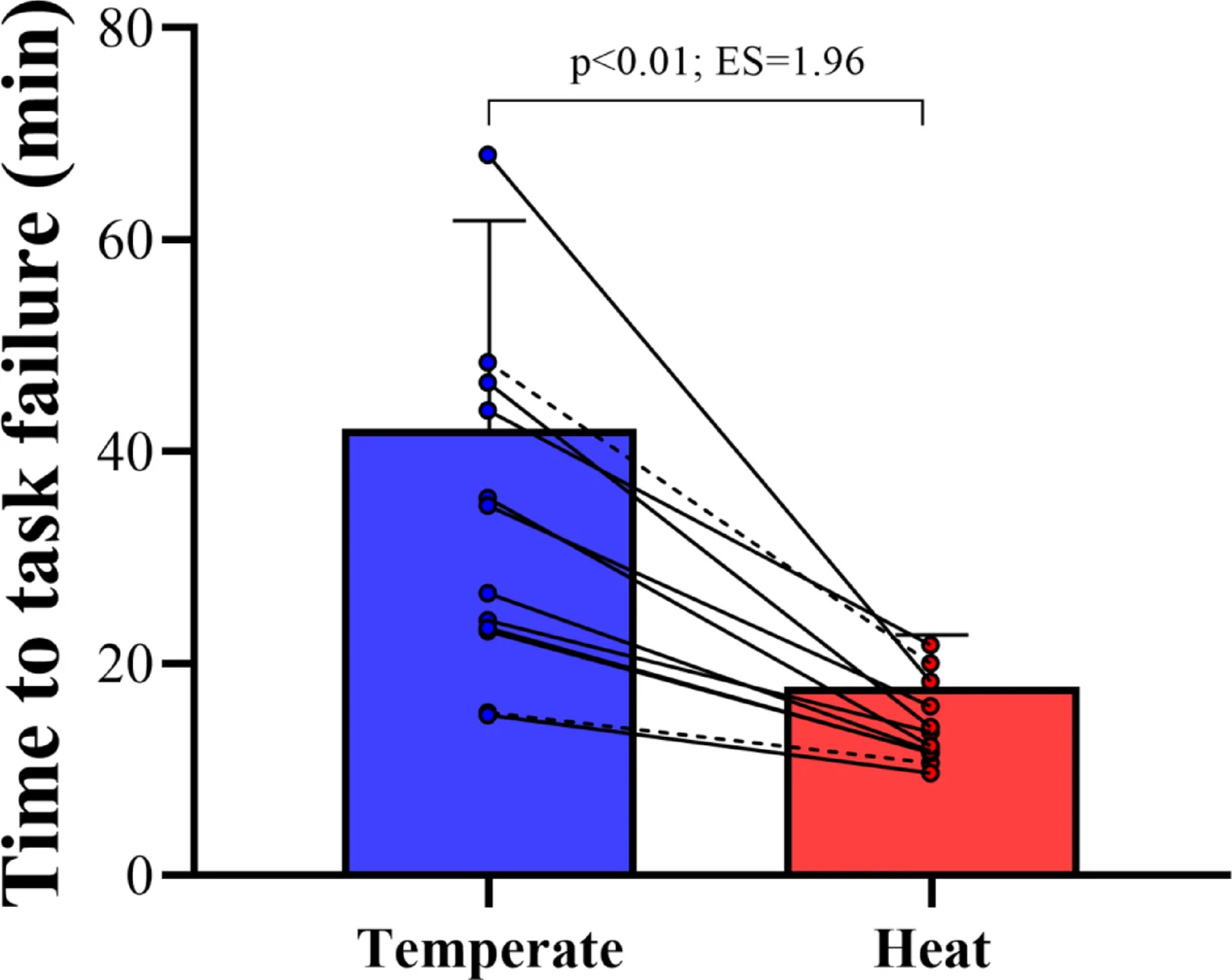
Time to task failure during treadmill running until task-failure at velocity associated with the respiratory compensation point. Temperate, exercise performed in a room at 22.4 °C; Heat, exercise performed in a room at 38.8 °C. *, different from temperate (paired *t* test); ES = effect size calculated as Cohen´s d. Dashed lines highlight females’ participants

**Fig. 3:**
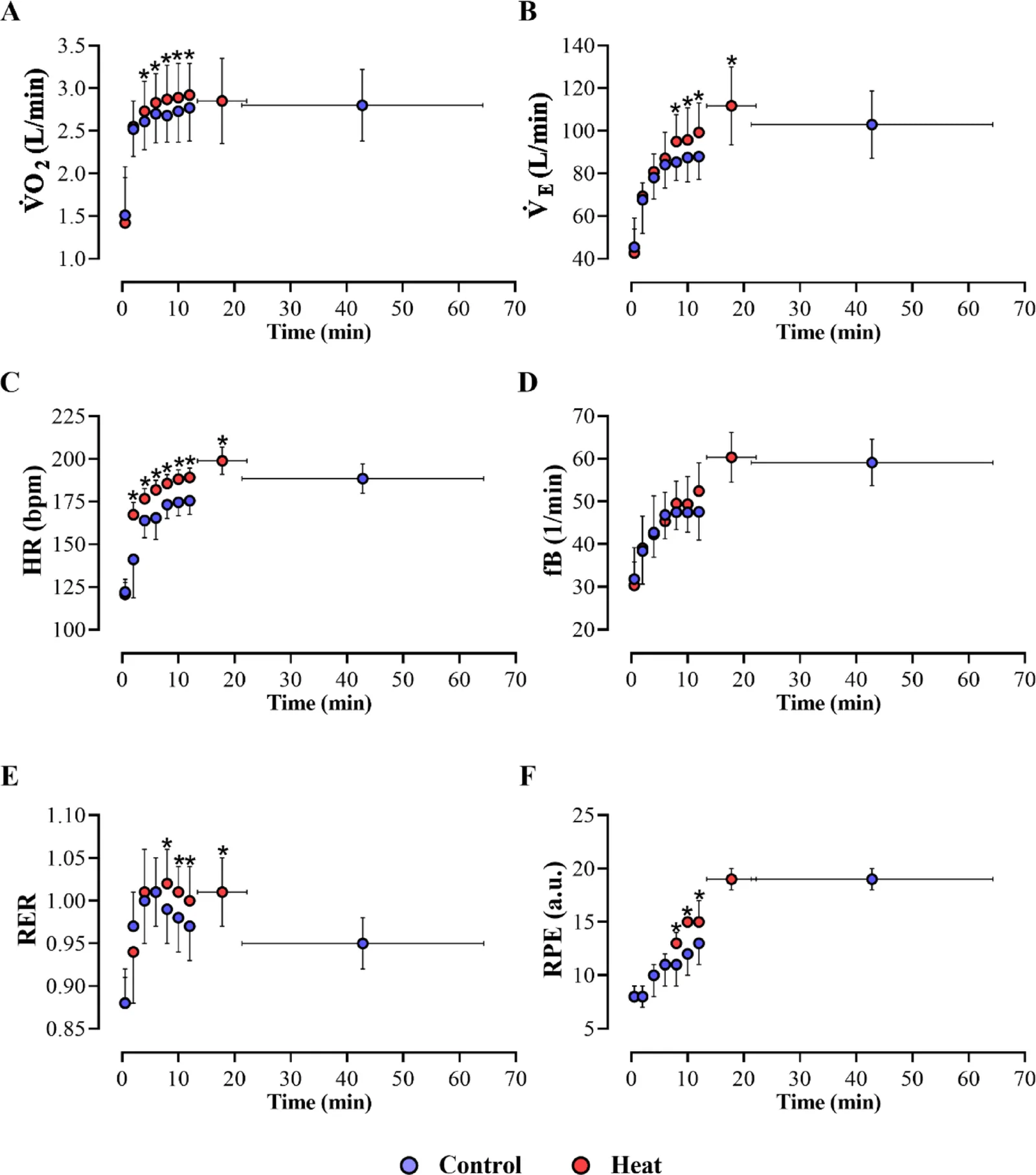
Exercise responses during running until task-failure at velocity associated with the respiratory compensation point. Temperate, exercise performed in a room at 22.4 °C; Heat, exercise performed in a room at 38.8 °C. V̇O_2_, rate of oxygen consumption (**A**); V̇E, ventilation (**B**); HR, heart rate (**C**); *f*B, breathing frequency (**D**); RER, respiratory exchange ratio (**E**); RPE, ratio of perceived exertion (**F**). Analyses were performed using the first 30 s of exercise, each 2 min until the shortest time to task failure evidenced among conditions (i.e., 12.05 min evidenced in the heat), and the 30 s at task failure. The statistical reports come from a two-way repeated measures ANOVA (condition × time). *, different from temperate in this time point (condition × interaction effect, post-hoc *p* < 0.05). Main effects of time and condition were not reported in the figure to improve clarity. *n* = 12 (crossover design)

The time to task failure was lower in the heat condition compared to the temperate condition (17.8 ± 4.4 min vs. 42.3 ± 20.1, *p* < 0.01, effect size = 1.96; Fig. 2), and this pattern was observed in both the female and male participants. The cardiorespiratory and RPE responses during exercise in the heat and temperate conditions are shown in Fig. 3A and F. Performing a matched duration comparison between conditions until the lower time to task failure recorded (i.e., 12.05 min in the heat), a significant time×condition interaction (*p* ≤ 0.04, F ≥ 2.82, $\:{\mathrm{n}}_{\mathrm{p}}^{\mathrm{2}}$≥0.20) was observed for V̇O_2_ (starting at 4 min of exercise)_,_ V̇_E_ (starting at 8 min of exercise), HR (starting at 2 min of exercise), RER (starting at 8 min of exercise), and RPE (starting at 8 min of exercise), with higher values during exercise in the heat condition compared to the temperate condition (post-hoc *p* ≤ 0.05).

### Neuromuscular function after exercise

**Fig. 4:**
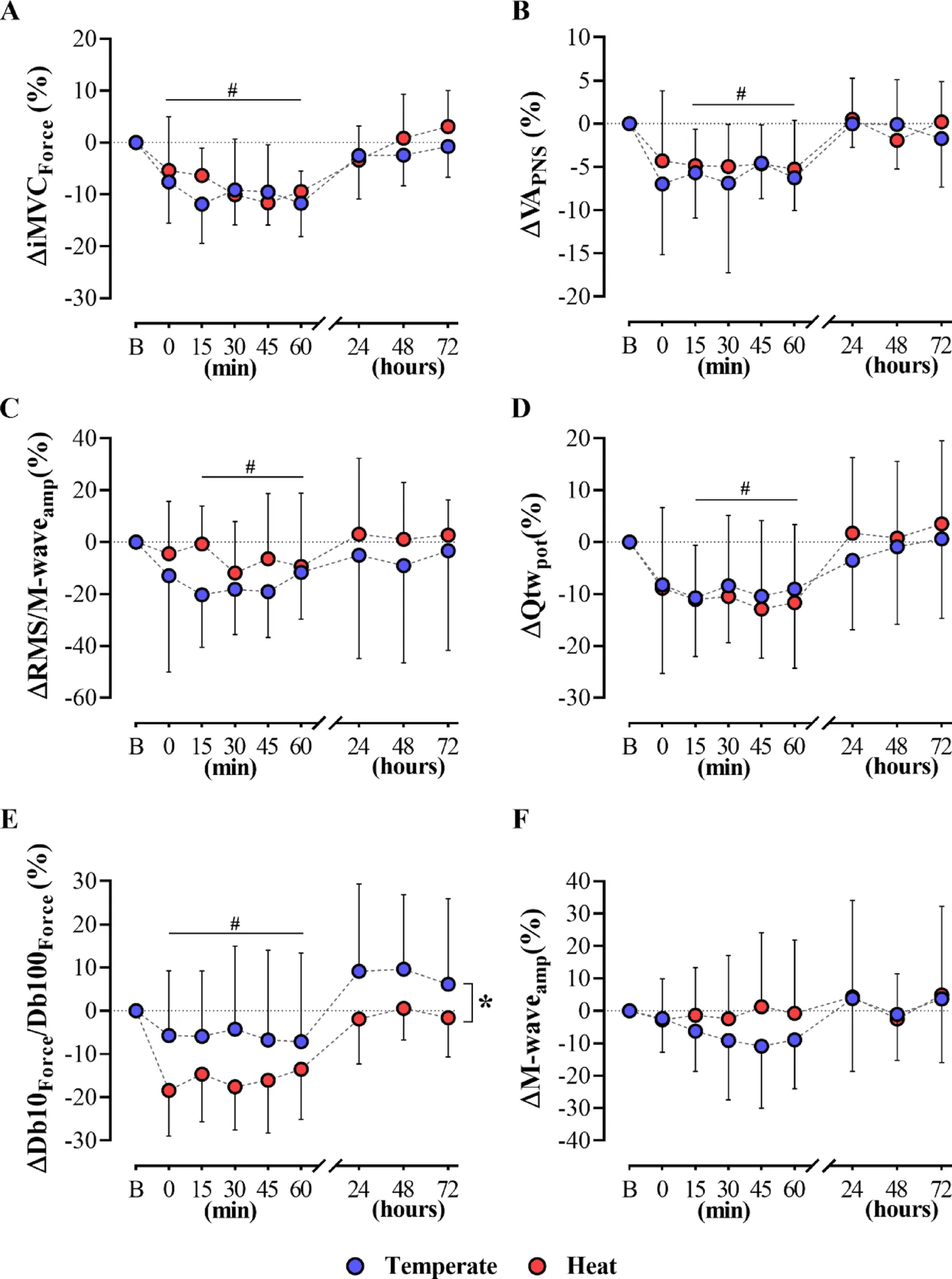
Percentage change from baseline in knee-extensors function outcomes. Temperate, exercise performed in a room at 22.4 °C; Heat, exercise performed in a room at 38.8 °C. B, baseline assessments; iMVC_Force_, highest voluntary force recorded during maximal voluntary isometric contractions of knee-extensors (**A**); VA_PNS_, voluntary activation measured through twitch interpolation technique (**B**); RMS/M-wave_amp_, ratio between the root mean square of electromyography signal from *vastus lateralis* during iMVC_Force_ and peak-to-peak M-wave amplitude ‒ M-wave_amp_ (**C**); Qtw_pot_, force evoked by a potentiated single stimulus at relaxed knee-extensors (**D**); Db10_Force_/Db100_Force_, ratio between the force evoked by a supramaximal high-frequency (100 Hz) and low-frequency (10 Hz) doublet stimulus at relaxed knee-extensors (**E**); M-wave_amp_ (**F**). The statistical reports come from a two-way repeated measures ANOVA. #, different from baseline (main effect of time, post hoc *p* < 0.05); *, different from temperate (main effect of condition, post-hoc *p* < 0.05). *n* = 12 (crossover design)

The changes in neuromuscular function following each exercise condition are displayed in Fig. 4A and 4F. A main effect of time was observed for iMVC_Force_ (*p* < 0.01, F = 21.57, $\:{\mathrm{n}}_{\mathrm{p}}^{\mathrm{2}}$=0.66), VA_PNS_ (*p* < 0.01, F = 8.21, $\:{\mathrm{n}}_{\mathrm{p}}^{\mathrm{2}}$=0.43), RMS/M-wave_amp_ (*p* = 0.03, F = 2.20, $\:{\mathrm{n}}_{\mathrm{p}}^{\mathrm{2}}$=0.17), Qtw_pot_ (*p* < 0.01, F = 8.44, $\:{\mathrm{n}}_{\mathrm{p}}^{\mathrm{2}}$=0.43), and Db10_Force_/Db100_Force_ (*p* < 0.01, F = 10.87, $\:{\mathrm{n}}_{\mathrm{p}}^{\mathrm{2}}$=0.49). Post-hoc analyses evidenced that these markers remained reduced from 15 to 60 min post-exercise (*p* ≤ 0.03), except for Db10_Force_/Db100_Force_, which was reduced immediately post-exercise and remained depressed until 60 min (post-hoc *p* ≤ 0.03). By 24 h post-exercise, all neuromuscular measures returned to baseline levels (p values for all post-hoc comparisons vs. baseline were > 0.05). However, over the 72-h monitoring period, a main effect of condition was observed for Db10_Force_/Db100_Force_ (*p* = 0.04, F = 5.00, $\:{\mathrm{n}}_{\mathrm{p}}^{\mathrm{2}}$=0.31), with a greater reduction following exercise in the heat (−9.24 ± 5.58%) compared to the temperate condition (−0.54 ± 13.6%; post-hoc *p* = 0.02). The two female participants exhibited a similar pattern of reduction in Db10_Force_/Db100_Force_ following exercise in the heat to that observed in the male participants. There were no time or condition effects on M-wave_amp_ (*p* ≥ 0.09, $\:{\mathrm{n}}_{\mathrm{p}}^{\mathrm{2}}$≤0.13). There were no condition × interaction effects for any of these outcomes (*p* ≥ 0.23, F ≤ 1.34, $\:{\mathrm{n}}_{\mathrm{p}}^{\mathrm{2}}$≤0.11).

### Heart rate variability after exercise

**Fig. 5:**
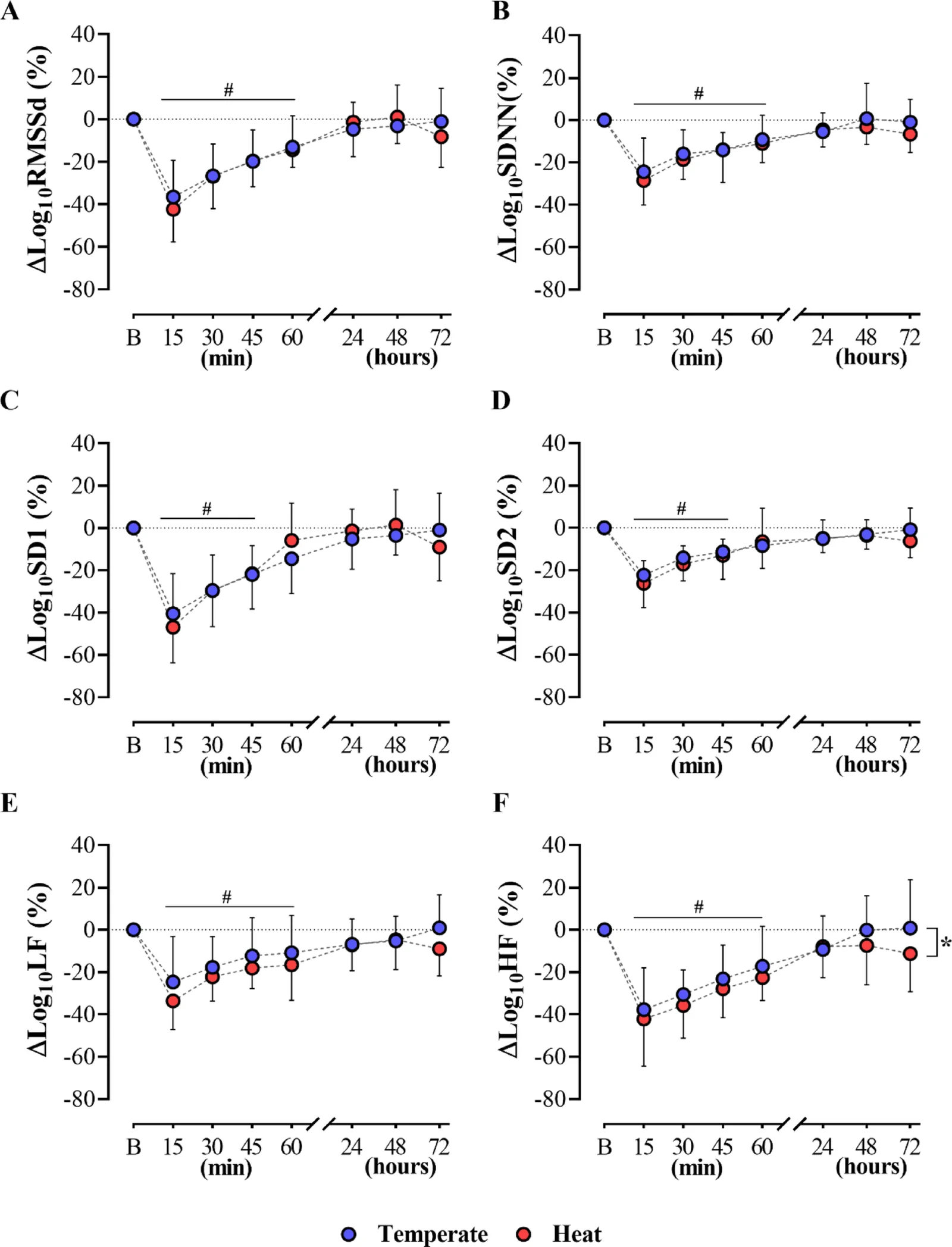
Percentage change from baseline in heart rate-variability outcomes. Temperate, exercise performed in a room at 22.4 °C; Heat, exercise performed in a room at 38.8 °C. B, baseline assessments; Log_10_RMSSD, base-ten logarithm of root-mean-square of successive differences of R-R intervals (**A**); Log_10_SDNN, base-ten logarithm of the standard deviation of N-N intervals (**B**); Log_10_SD1, base-ten of the standard deviation perpendicular to the line of identity (**C**); Log_10_SD2, base-ten of the standard deviation parallel to the line of identity (**D**); Log_10_LF, base-ten low-frequency power (**E**); Log_10_HF, base-ten high-frequency power (**F**). The statistical reports come from a two-way repeated measures ANOVA. #, different from baseline (main effect of time, post-hoc *p* < 0.05); *, different from temperate (main effect of condition, post-hoc *p* < 0.05). *n* = 12 (crossover design)

The changes in HRV metrics following each exercise condition are presented in Fig. 5A and F. A main effect of time was evidenced for Log_10_RMSSD (*p* < 0.01, F = 28.08, $\:{\mathrm{n}}_{\mathrm{p}}^{\mathrm{2}}$=0.72), Log_10_SDNN (*p* < 0.01, F = 20.23, $\:{\mathrm{n}}_{\mathrm{p}}^{\mathrm{2}}$=0.65), Log_10_SD1 (*p* < 0.01, F = 20.22, $\:{\mathrm{n}}_{\mathrm{p}}^{\mathrm{2}}$=0.65), Log_10_SD2 (*p* < 0.01, F = 17.98, $\:{\mathrm{n}}_{\mathrm{p}}^{\mathrm{2}}$=0.62), Log_10_LF (*p* < 0.01, F = 17.07, $\:{\mathrm{n}}_{\mathrm{p}}^{\mathrm{2}}$=0.60), and Log_10_HF (*p* < 0.01, F = 20.11, $\:{\mathrm{n}}_{\mathrm{p}}^{\mathrm{2}}$=0.66). All metrics remained reduced from 15 min post-exercise until 45–60 min post-exercise (post-hoc *p* ≤ 0.02). By 24 h post-exercise, all values returned to baseline levels (*p* > 0.05 for all post-hoc comparisons vs. baseline). Over the 72-h recovery period, a main effect of condition was observed for Log_10_HF (*p* = 0.04, F = 5.01, $\:{\mathrm{n}}_{\mathrm{p}}^{\mathrm{2}}$=0.31), with a greater post-exercise reduction in the heat (−22.11 ± 11.15%) compared to the temperate condition (−16.75 ± 10.34%; post-hoc *p* = 0.02). There were no condition × interaction effects for any of these outcomes (*p* ≥ 0.29, F ≤ 1.27, $\:{\mathrm{n}}_{\mathrm{p}}^{\mathrm{2}}$≤0.10). The HRV responses displayed similar patterns for both the female and male participants.

### Plasma markers of muscle microtrauma

The changes in the concentration of plasma markers of muscle microtrauma post-exercise in both conditions are presented in Table 2. A main effect of time was observed for both plasma CK (*p* < 0.01, F = 7.99, $\:{\mathrm{n}}_{\mathrm{p}}^{\mathrm{2}}$=0.42) and LDH (*p* = 0.01, F = 5.90, $\:{\mathrm{n}}_{\mathrm{p}}^{\mathrm{2}}$=0.35). Post-hoc analyses evidenced that plasma CK increased immediately post-exercise (0.5-h) and remained elevated at 24-h post-exercise (post-hoc *p* ≤ 0.04), while LDH increased only at 24-h post-exercise (post-hoc *p* = 0.04). A condition×time interaction effect was observed for CK (*p* = 0.03, F = 2.97, $\:{\mathrm{n}}_{\mathrm{p}}^{\mathrm{2}}$=0.21). Post-hoc comparisons indicated that plasma CK was lower in the heat condition compared to the temperate condition at 24-h post-exercise (*p* = 0.03). Notably, the percentage difference between conditions (i.e., heat-condition change from baseline *minus* temperate-condition change from baseline) in the 24-h post-exercise plasma CK was − 55.4 ± 65.6%, whereas the percentage difference between conditions in the time to task failure was − 53.2 ± 12.7%. The correlation between these variables was significant and very large (*r* = 0.75, *p* < 0.01). No other condition effects or condition×time interactions were observed for plasma CK or LDH (*p* ≤ 0.06, F ≤ 4.27, $\:{\mathrm{n}}_{\mathrm{p}}^{\mathrm{2}}$≤0.28).

**Table 2:** Percentage changes from baseline (i.e., 0%) in plasma markers of muscle microtrauma after each exercise condition

		Time post-exercise			
		*0.5-h*	*24-h*	*48-h*	*72-h*
CK (Δ% from baseline)	Temperate	37.1 ± 30.1^a^	131.1 ± 142.5^a&^	36.5 ± 60.9	40.4 ± 85.7
	Heat	26.2 ± 51.9 ^a^	35.3 ± 41.4 ^a^	9.1 ± 35.1	12.4 ± 49.8
LDH (Δ% from baseline)	Temperate	22.01 ± 39.3	27.4 ± 26.8^a^	−6.6 ± 18.5	15.5 ± 44.6
	Heat	8.9 ± 26.3	2.6 ± 14.4^a^	−1.2 ± 17.2	15.5 ± 30.1

Data are presented as mean ± standard deviation (range). *CK*: plasma creatine kinase concentration; *LDH*: plasma lactate dehydrogenase concentration. ^a^*p*<0.05, different from rest (main time effect in a two-way ANOVA, post-hoc *p* < 0.05); ^&^*p* < 0.05, different from heat at this time point (condition × time interaction effect in a two-way ANOVA, post-hoc *p* < 0.05)

## Discussion

This study investigated the effects of running in the heat on time to task failure during a fixed workload (i.e., vRCP measure in a temperate environment) and post-exercise neuromuscular function, cardiac autonomic modulation, and plasma markers of muscle microtrauma. Key findings demonstrated that running in the heat exacerbated low-frequency force depression and Log_10_HF withdrawal over 72 h post-exercise, without influencing other neuromuscular or HRV-derived outcomes. Notably, these impairments occurred despite a ~ 53% reduction in time to task failure compared to the temperate condition. Interestingly, plasma CK concentrations were lower in the heat at 24-h post-exercise, and the between-condition differences in plasma CK at this time point were strongly associated with the between-condition difference in time to task failure (*r* = 0.75). Although causality cannot be established, this finding suggests that the shorter exercise duration in the heat may have contributed to the attenuated CK response. These findings highlight that running in the heat until task failure exerts divergent effects on post-exercise responses. Specifically, it exacerbated the decline in low-frequency force production and Log_10_HF, an HRV-derived indicator of cardiac vagal modulation, while attenuating the increase in a plasma marker of muscle microtrauma. Consequently, practitioners should consider assessing low-frequency force production (Ridard et al. 2022) and cardiac vagal modulation (Stanley et al. 2013) to more accurately assess disturbances specific to this scenario. Furthermore, when implementing recovery strategies, methods that mitigate low-frequency force depression (Chaillou and Cheng 2019) and cardiac vagal withdrawal (Stanley et al. 2013) should be prioritized over those targeting muscle microtrauma (Malta et al. 2018). From a training perspective (Philp et al. 2022), the present findings suggest that athletes may require additional recovery of neuromuscular and autonomic function following running in the heat, even when exercise duration is substantially reduced. Therefore, practitioners should consider adjusting the timing, intensity, and neuromuscular demands of subsequent training sessions to account for the greater impairments in low-frequency force production and HRV indicators of cardiac vagal modulation observed after exercise in the heat.

The observed reduction in time to task failure aligns with previous studies on locomotor efforts in hot environments (de Korte et al. 2021a; Júnior et al. 2023; Nybo and Nielsen 2001; Périard et al. 2011). As previously suggested, a gradual increase in thermal strain (i.e., rise in whole body temperature) elevates the relative exercise intensity for any given work rate during CWR in the heat (Périard et al. 2021; Schlader et al. 2011). In the present study, matched duration comparisons evidenced higher V̇O_2_, V̇_E_, RER, and HR during exercise in the heat compared to the temperate condition. These findings suggest greater metabolic and cardiorespiratory strain for the prescribed absolute work rate (i.e., vRCP measured in a temperate environment) (Périard et al. 2021). This greater strain was accompanied by increased perceived exertion. Collectively, these findings suggest that heightened inhibitory afferent feedback from peripheral organs such as the heart, active skeletal musculature, and respiratory muscles, combined with greater perception of exertion, may have contributed to the earlier cessation of exercise in the heat (Périard et al. 2021). This mechanism appears to serve as a protective response that prevents catastrophic homeostatic disturbance during exercise (Noakes 2000; Noakes et al. 2005). Notably, the heat-induced impairment in time to task failure observed in the current study (effect size = 1.96) was greater than previously reported (effect size = 1.33) (de Korte et al. 2021a), yet lower than that observed by others (ES ranging from 2.9 to 6.3) (Galloway and Maughan 1997; Mitchell et al. 2014). Differences in exercise protocols (e.g., CWR vs. incremental), intensity (i.e., relative workload to V̇O_2max_), and environmental conditions (i.e., laboratory temperature) likely account for these discrepancies.

Despite reduced time to task failure, the hot environment did not influence the time course or magnitude of decline in knee-extensor force-generating capacity (iMVC_Force_), muscle activation (i.e., VA_PNS_), or contractile function (i.e., Qtw_pot_) for up to 72-h post-exercise. These findings extend previous observations in semiprofessional soccer players (Nybo et al. 2013), suggesting that in recreationally trained individuals, heat exposure does not influence these impairments following continuous running at heavy domain, even within the first hour post-exercise. However, running in the heat exacerbated the accumulated reduction in the Db10_Force_/Db100_Force_ ratio over 72-h post-exercise, a finding that indicates more pronounced low-frequency force depression (Jones 1996). Although it does not compromise maximal force-generating capacity, low-frequency force depression is linked to increased central drive and perceived effort during subsequent tasks (Keeton and Binder-Macleod 2006). This impairment of neuromuscular function is primarily attributed to reduced Ca^++^ release from the sarcoplasmic reticulum (Jones 1996). Potential mechanisms underlying the exacerbated impairment after exercising in hot environments include hyperthermia-induced production of reactive oxygen/nitrogen species (de Melo-Marins et al. 2021). Although mechanistic assessments were not conducted in this study, the findings are the first to demonstrate that monitoring low-frequency force depression, instead of other neuromuscular function markers (e.g., peak voluntary force, voluntary activation), can be useful for identifying specific heat-related neuromuscular disturbances.

Running in the heat also induced a greater accumulated decrease in HRV-derived Log_10_HF over the 72-h recovery period. Because Log_10_HF reflects vagal activity (Shaffer and Ginsberg 2017), this finding indicates a more pronounced reduction in cardiac vagal modulation after exercise in the heat. Previous studies have shown that heat exposure during exercise induced greater cardiac vagal withdrawal within the first 10 to 45 min of recovery compared to temperate and dry heat conditions (Brenner et al. 1997; Peçanha et al. 2017). The present findings extend this evidence by showing that this exacerbated suppression may persist for several days. Although cardiac vagal withdrawal for days post-exercise is likely multifactorial and may be influenced by heat-related reductions in plasma volume (Stanley et al. 2013), a mechanistic assessment of the factors underlying the exacerbated withdrawal observed after running in the heat was beyond the scope of this study. Notably, Log_10_HF was more sensitive than the other HRV metrics in detecting heat-related alterations in vagal modulation. Given that frequency domain metrics are considered more suitable for inferring vagal activity from short-term recordings (~ 10 min) (Task Force 1996). These findings reinforce this recommendation and suggest that Log_10_HF, rather than HRV-derived time-domain measurements (e.g., RMSSd, SDNN), may be preferred for tracking cardiac vagal modulation after exercising in the heat.

In addition, running in the heat attenuated the increase in plasma CK observed 24 h post-exercise. The significant large correlation (*r* = 0.75) between the percentage differences in plasma CK response and time to task failure across conditions suggests that the attenuated CK response may be related, at least in part, to the shorter exercise duration achieved in the heat. Although hyperthermia has been suggested to exacerbate muscle fiber disruption during exercise in the heat (Roberts et al. 2023), our findings align with previous observations in semiprofessional soccer players (Nybo et al. 2013), where heat exposure during a match limited the post-exercise CK increase, likely due to reduced exercise volume (i.e., total distance covered). Our findings extend this observation to recreationally trained individuals performing continuous endurance running at the upper boundary of the heavy domain. Thus, while heat stress alone may exacerbate exercise-induced disruption of muscle integrity (Roberts et al. 2023), the concomitant reduction in exercise volume appears to exert greater influence on the extent of muscle microtrauma observed post-exercise. Therefore, combining exercise volume indicators (e.g., total duration, distance covered) with plasma makers such as CK is essential for evaluating the extent of muscle microtrauma after endurance exercise in the heat.

We acknowledge several limitations of the present study. First, core and muscle temperature, as well as sweat and fluid losses, were not measured during or after exercise. Although these variables may have provided additional mechanistic insight, their absence does not preclude interpretation of the observed changes in neuromuscular function, HRV-derived outcomes, and plasma markers of muscle microtrauma. Second, although a constant-workload protocol was selected to minimize the confounding effects of pacing strategy and facilitate direct comparisons between environmental conditions (Lander et al. 2009), heat stress may alter exercise thresholds (de Barros et al., 2011; Maunder et al. 2021). However, previous evidence suggests that the respiratory compensation point and the workload at which it occurs are minimally influenced by heat exposure (Smolander et al. 1986; Bourgois et al. 2023). Investigating the influence of heat stress on fixed external demands likely enhances the practical applicability of our findings, as individuals in real-world training and competition typically strive to maintain planned exercise demand. Third, because the study did not include a temperate-condition trial matched in duration to the heat-condition trial, the isolated effects of heat exposure cannot be distinguished from those attributable to differences in exercise duration. Future studies employing duration-matched exercise protocols are warranted to address this issue. Fourth, CO₂ accumulation within the environmental chamber was not directly controlled and should therefore be considered when interpreting the respiratory measurements. Finally, the present investigation was not designed or adequately powered to examine sex-related differences. Although we attempted to recruit a balanced number of females, our screening process revealed that our study design, which involved multiple peripheral nerve stimulations, did not attract many interested females. Nonetheless, we considered it appropriate to retain the interested female participants (*n* = 2) in our sample to encourage future female participation in research of this kind (Smith et al. 2025). Thus, caution is warranted when generalizing these findings, particularly to female populations. Because of known sex-related differences in thermoregulation and fatigability (Yanovich et al. 2020; Ansdell et al. 2020), future studies should include a larger and more balanced representation of female participants.

## Conclusions

For the same absolute workload (i.e., vRCP measured in a temperate environment), exercising in the heat reduced running time to task failure by approximately 53%. Despite this shorter exercise duration, running in the heat exacerbated low-frequency force depression and produced a greater reduction in Log_10_HF over 72-h post-exercise, without influencing the remaining neuromuscular function or HRV-derived outcomes. In contrast, running in the heat attenuated the increase in plasma CK observed 24 h post-exercise. The significant correlation between the condition-related differences in plasma CK and time to task failure suggests that the attenuated CK response may be related, at least in part, to the shorter exercise duration achieved in the heat. Collectively, these findings indicate that running in the heat not only impairs endurance performance but also produces divergent effects on post-exercise responses. From an applied perspective, practitioners should consider monitoring low-frequency force production and cardiac vagal modulation following exercise in the heat and prioritize recovery strategies that target these physiological disturbances.

## Supplementary Information

Below is the link to the electronic supplementary material.

Supplementary Material 1

## Data Availability

The datasets generated during and/or analyzed during the current study are available from the corresponding author on reasonable request.

## Abbreviations

ANOVA: Analysis of variance
CK: Plasma creatine kinase concentration
CWR: Constant work-rate
Db100_Force_: Force evoked by the doublet high-frequency stimuli
Db10_Force_: Force evoked by the doublet low-frequency stimuli
Db10_Force_/Db100_Force_: The ratio between the force evoked by the doublet low-frequency stimuli and doublet low-frequency stimuli
EMG: Surface electromyography activity
*f*B: Breathing frequency
GXT: Graded exercise test
HF: High-frequency power
HR: Heart rate
HRV: Heart rate variability
iMVC_Force_: Peak voluntary force during isometric maximal voluntary contractions
iMVCs: Isometric maximal voluntary contractions
LDH: Plasma lactate dehydrogenase concentration
LF: Low-frequency power
Log10: Base-10 log-transformed
PetCO_2_: End-tidal pressure of CO_2_
PNS: Peripheral nerve stimulation
Qtw_pot_: Potentiated peak force evoked by single stimulus
RCP: Respiratory compensation point
RER: Respiratory exchange ratio
RMS/M-wave_amp_: The ratio between the root mean square of the *vastus lateralis *surface electromyography activity and the M-wave amplitude
RMSSD: Root-mean-square of successive differences of R-R intervals
RMS_VL_: Root mean square of the *vastus lateralis* surface electromyography activity signal
RPE: Rate of perceived exertion
SD1: The standard deviation perpendicular to the line of identity
SD2: The standard deviation parallel to the line of identity
SDNN: The standard deviation of N-N intervals
VA_PNS_: Voluntary activation
V̇CO_2_: Carbon dioxide output
V̇_E_: Ventilation
V̇_E_/ V̇CO_2_: Ratio between ventilation and carbon dioxide output
V̇O_2_: Oxygen uptake
V̇O_2max_: Maximal oxygen uptake
vRCP: Velocity corresponding to respiratory compensation point
vV̇O_2max_: Velocity corresponding to maximal oxygen uptake
