# Supplementary material for: Running in the heat reduces time to task failure, attenuates biomarkers of muscle microtrauma, and exacerbates low-frequency force depression and cardiac vagal withdrawal over 72-h post-exercise
Source: Eur J Appl Physiol. 2026 Jul 25;126(10):5675–90. doi: 10.1007/s00421-026-06367-7 (PMC13634002; doi:10.1007/s00421-026-06367-7)
Supplement: Supplementary file 1 [file 421_2026_6367_MOESM1_ESM.docx]

**Supplemental data file for the manuscript:** Running in the heat reduces time to task failure, attenuates biomarkers of muscle microtrauma, and exacerbates low-frequency force depression and cardiac vagal withdrawal over 72-h post-exercise

**Summary**

Raw data of study ………………………………………………………………………………………………………... 2

**Raw data of the study**

**Table 1.** Descriptive data related to the knee-extensors outcomes evaluated in the study.

|  |  | **Baseline** | **0min post-exercise** | **15min post-exercise** | **30min post-exercise** | **45min post-exercise** | **60min post-exercise** | **24h post-exercise** | **48h post-exercise** | **72h post-exercise** |
| --- | --- | --- | --- | --- | --- | --- | --- | --- | --- | --- |
| **iMVC_Force_** (N) | TEMP | 586.3 ± 106.4  (526.1 to 646.5) | 542.8 ± 111.6  (479.6 to 606.0) | 526.3 ± 108.8  (458.8 to 593.7) | 529.9 ± 90.6  (478.6 to 581.1) | 527.4 ± 88.6  (477.3 to 577.6) | 516.2 ± 88.8  (465.9 to 566.4) | 571.3 ± 105.1  (511.8 to 630.8) | 572.6 ± 109.8  (510.4 to 634.7) | 579.7 ± 94.0  (526.5 to 632.9) |
|  | HEAT | 578.6 ± 133.2  (503.2 to 654.0) | 541.5 ± 117.6  (474.9 to 608.1) | 547.0 ± 120.3  (472.5 to 621.6) | 518.2 ± 135.2  (438.3 to 598.1) | 509.4 ± 115.3  (444.2 to 574.6) | 522.9 ± 119.0  (455.5 to 590.2) | 556.3 ± 130.6  (482.4 to 630.2) | 582.4 ± 134.4  (506.4 to 658.5) | 602.3 ± 144.5  (516.9 to 687.7) |
|  |  |  |  |  |  |  |  |  |  |  |
| **VA_PNS_** (%) | TEMP | 89.5 ± 8.4  (84.8 to 94.3) | 83.2 ± 9.9  (77.6 to 88.8) | 83.9 ± 10.2  (77.6 to 90.2) | 83.4 ± 11.8  (76.7 to 90.0) | 85.5 ± 9.1  (80.4 to 90.6) | 83.9 ± 8.9  (78.9 to 89.0) | 89.5 ± 8.3  (84.8 to 94.2) | 89.5 ± 9.5  (84.1 to 94.9) | 87.9 ± 9.0  (82.9 to 93.0) |
|  | HEAT | 89.7 ± 8.5  (84.9 to 94.5) | 85.5 ± 7.7  (81.2 to 89.9) | 84.7 ± 8.4  (79.5 to 89.9) | 85.2 ± 9.8  (79.4 to 91.0) | 85.5 ± 8.3  (80.8 to 90.2) | 85.0 ± 8.9  (79.9 to 90.0) | 89.9 ± 6.6  (86.2 to 93.6) | 87.9 ± 8.1  (83.3 to 92.5) | 89.6 ± 10.6  (83.3 to 95.8) |
|  |  |  |  |  |  |  |  |  |  |  |
| **RMS_VL_/ M-wave_amp_** | TEMP | 0.07 ± 0.08  (0.03 to 0.12) | 0.05 ± 0.01  (0.04 to 0.05) | 0.06 ± 0.05  (0.03 to 0.08) | 0.06 ± 0.07  (0.02 to 0.10) | 0.06 ± 0.04  (0.03 to 0.08) | 0.07 ± 0.09  (0.02 to 0.12) | 0.05 ± 0.02  (0.04 to 0.07) | 0.05 ± 0.01  (0.04 to 0.06) | 0.05 ± 0.01  (0.05 to 0.06) |
|  | HEAT | 0.06 ± 0.01  (0.04 to 0.06) | 0.06 ± 0.03  (0.04 to 0.08) | 0.05 ± 0.02  (0.04 to 0.06) | 0.05 ± 0.01  (0.04 to 0.05) | 0.05 ± 0.02  (0.04 to 0.06) | 0.04 ± 0.02  (0.04 to 0.05) | 0.05 ± 0.02  (0.04 to 0.06) | 0.05 ± 0.01  (0.04 to 0.06) | 0.06 ± 0.01  (0.05 to 0.07) |
|  |  |  |  |  |  |  |  |  |  |  |
| ***Q*tw** (N) | TEMP | 196.1 ± 40.3  (173.2 to 218.9) | 177.9 ± 42.0  (154.1 to 201.7) | 178.4 ± 28.0  (161.0 to 195.7) | 177.8 ± 36.3  (157.3 to 198.4) | 173.3 ± 35.0  (153.5 to 193.1) | 176.8 ± 36.9  (156.0 to 197.7) | 187.4 ± 36.6  (166.7 to 208.1) | 192.6 ± 40.8  (169.5 to 215.6) | 195.3 ± 39.3  (173.0 to 217.5) |
|  | HEAT | 199.3 ± 51.3  (170.3 to 228.3) | 178.2 ± 42.8  (154.0 to 202.4) | 185.9 ± 34.0  (164.8 to 202.2) | 176.0 ± 44.4  (149.8 to 202.2) | 171.5 ± 39.5  (149.2 to 193.8) | 172.7 ± 37.8  (151.4 to 194.1) | 198.9 ± 43.4  (174.3 to 223.4) | 196.0 ± 41.4  (172.6 to 219.5) | 209.3 ± 32.8  (189.9 to 228.7) |
|  |  |  |  |  |  |  |  |  |  |  |
| **Db_10Force_/Db_100Force_** | TEMP | 0.93 ± 0.17  (0.84 to 1.03) | 0.87 ± 0.19  (0.77 to 0.98) | 0.86 ± 0.07  (0.81 to 0.90) | 0.87 ± 0.12  (0.80 to 0.94) | 0.85 ± 0.14  (0.77 to 0.92) | 0.84 ± 0.13  (0.77 to 0.92) | 0.99 ± 0.09  (0.94 to 1.04) | 1.00 ± 0.07  (0.96 to 1.04) | 0.96 ± 0.09  (0.91 to 1.01) |
|  | HEAT | 0.98 ± 0.10  (0.92 to 1.03) | 0.80 ± 0.14  (0.72 to 0.88) | 0.83 ± 0.13  (0.75 to 0.90) | 0.80 ± 0.11  (0.73 to 0.86) | 0.81 ± 0.11  (0.75 to 0.86) | 0.84 ± 0.12  (0.78 to 0.91) | 0.95 ± 0.12  (0.89 to 1.02) | 0.98 ± 0.10  (0.92 to 1.03) | 0.96 ± 0.08  (0.91 to 1.00) |
|  |  |  |  |  |  |  |  |  |  |  |
| **M-wave_amp_** (mV) | TEMP | 12.4 ± 4.4  (9.9 to 14.9) | 12.5 ± 5.5  (9.2 to 15.7) | 11.6 ± 4.0  (9.1 to 14.1) | 11.2 ± 4.1  (8.8 to 13.6) | 10.8 ± 4.1  (8.3 to 13.2) | 11.0 ± 4.3  (8.5 to 13.6) | 13.9 ± 3.6  (11.9 to 16.0) | 13.3 ± 3.4  (11.4 to 15.3) | 13.7 ± 3.3  (11.8 to 15.7) |
|  | HEAT | 12.8 ± 3.5  (10.8 to 14.8) | 11.8 ± 3.8  (9.7 to 13.9) | 11.3 ± 4.4  (8.6 to 14.1) | 11.4 ± 3.0  (9.7 to 13.2) | 12.1 ± 4.2  (9.6 to 14.6) | 11.9 ± 3.8  (9.6 to 14.1) | 13.3 ± 3.2  (11.5 to 15.1) | 12.5 ± 2.8  (10.9 to 14.1) | 14.2 ± 3.2  (12.2 to 16.2) |

Mean ± SD (95%CI). TEMP, temperate condition in which exercise was performed in a room at 22.4°C; Heat, exercise performed in a room at 38.8°C. iMVC_Force_, highest voluntary force recorded during maximal voluntary isometric contractions of knee-extensors; VA_PNS_, voluntary activation measured through twitch interpolation technique; RMS/M-wave_amp_, ratio between the root mean square of electromyography signal from *vastus lateralis* during iMVC_Force_ and peak-to-peak M-wave amplitude ‒ M-wave_amp_; Qtw_pot_, force evoked by a potentiated single stimulus at relaxed knee-extensors; Db10_Force_/Db100_Force_, ratio between the force evoked by a supramaximal high-frequency (100Hz) and low-frequency (10 Hz) doublet stimulus at relaxed knee-extensors; M-wave_amp_. N=12(crossover design).

**Table 2.** Descriptive data related to the heart rate variability metrics evaluated in the study.

|  |  | **Baseline** | **15min post-exercise** | **30min post-exercise** | **45min post-exercise** | **60min post-exercise** | **24h post-exercise** | **48h post-exercise** | **72h post-exercise** |
| --- | --- | --- | --- | --- | --- | --- | --- | --- | --- |
| **Log_10_RMSSD** (ms) | TEMP | 1.58 ± 0.22  (1.46 to 1.70) | 1.00 ± 0.36  (0.77 to 1.22) | 1.16 ± 0.31  (0.99 to 1.34) | 1.27 ± 0.29  (11.10 to 1.43) | 1.37 ± 0.31  (1.20 to 1.55) | 1.49 ± 0.21  (1.38 to 1.61) | 1.53 ± 0.21  (1.41 to 1.64) | 1.52 ± 0.20  (1.40 to 1.64) |
|  | HEAT | 1.59 ± 0.20  (1.47 to 1.70) | 0.90 ± 0.30  (0.72 to 1.08) | 1.18 ± 0.30  (1.01 to 1.36) | 1.28 ± 0.27  (1.12 to 1.43) | 1.36 ± 0.24  (1.23 to 1.50) | 1.56 ± 0.23  (1.43 to 1.70) | 1.59 ± 0.20  (1.47 to 1.70) | 1.45 ± 0.28  (1.30 to 1.61) |
|  |  |  |  |  |  |  |  |  |  |
| **Log_10_SDNN** (ms) | TEMP | 1.79 ± 0.17  (1.69 to 1.89) | 1.36 ± 0.31  (1.17 to 1.55) | 1.50 ± 0.22  (1.37 to 1.63) | 1.53 ± 0.27  (1.38 to 1.68) | 1.62 ± 0.23  (1.49 to 1.75) | 1.69 ± 0.15  (1.61 to 1.77) | 1.80 ± 0.35  (1.61 to 2.00) | 1.75 ± 0.13  (1.68 to 1.82) |
|  | HEAT | 1.84 ± 0.17  (1.74 to 1.93) | 1.29 ± 0.23  (1.16 to 1.43) | 1.52 ± 0.21  (1.39 to 1.64) | 1.57 ± 0.18  (1.47 to 1.68) | 1.63 ± 0.17  (1.53 to 1.72) | 1.75 ± 0.16  (1.66 to 1.84) | 1.77 ± 0.13  (1.69 to 1.84) | 1.71 ± 0.18  (1.61 to 1.81) |
|  |  |  |  |  |  |  |  |  |  |
| **Log_10_SD1** (ms) | TEMP | 1.43 ± 0.22  (1.31 to 1.55) | 0.85 ± 0.36  (0.62 to 1.07) | 1.01 ± 0.31  (0.84 to 1.19) | 1.12 ± 0.29  (0.95 to 1.28) | 1.23 ± 0.31  (1.05 to 1.40) | 1.34 ± 0.21  (1.23 to 1.46) | 1.38 ± 0.21  (1.26 to 1.49) | 1.37 ± 0.20  (1.25 to 1.49) |
|  | HEAT | 1.43 ± 0.20  (1.32 to 1.55) | 0.75 ± 0.30  (0.57 to 0.93) | 1.03 ± 0.30  (0.86 to 1.21) | 1.13 ± 0.27  (0.98 to 1.28) | 1.38 ± 0.72  (0.97 to 1.78) | 1.41 ± 0.23  (1.28 to 1.55) | 1.44 ± 0.20  (1.32 to 1.55) | 1.30 ± 0.28  (1.15 to 1.46) |
|  |  |  |  |  |  |  |  |  |  |
| **Log_10_SD2** (ms) | TEMP | 1.91 ± 0.17  (1.82 to 2.01) | 1.50 ± 0.31  (1.31 to 1.69) | 1.64 ± 0.22  (1.51 to 1.76) | 1.69 ± 0.23  (1.56 to 1.82) | 1.75 ± 0.22  (1.63 to 1.88) | 1.81 ± 0.15  (1.73 to 1.89) | 1.85 ± 0.15  (1.77 to 1.93) | 1.88 ± 0.12  (1.81 to 1.95) |
|  | HEAT | 1.97 ± 0.17  (1.87 to 2.06) | 1.43 ± 0.23  (1.30 to 1.57) | 1.65 ± 0.20  (1.53 to 1.77) | 1.71 ± 0.17  (1.61 to 1.81) | 1.84 ± 0.39  (1.62 to 2.06) | 1.86 ± 0.17  (1.77 to 1.96) | 1.89 ± 0.13  (1.81 to 1.96) | 1.84 ± 0.17  (1.74 to 1.93) |
|  |  |  |  |  |  |  |  |  |  |
| **Log_10_LF** (ms^2^) | TEMP | 3.27 ± 0.46  (3.01 to 3.53) | 2.47 ± 0.70  (2.03 to 2.90) | 2.66 ± 0.43  (2.42 to 2.90) | 2.83 ± 0.53  (2.53 to 3.13) | 2.87 ± 0.47  (2.61 to 3.14) | 3.01 ± 0.33  (2.83 to 3.20) | 3.06 ± 0.34  (2.87 to 3.25) | 3.24 ± 0.36  (3.03 to 3.45) |
|  | HEAT | 3.38 ± 0.40  (3.16 to 3.61) | 2.22 ± 0.57  (1.88 to 2.55) | 2.65 ± 0.46  (2.38 to 2.93) | 2.77 ± 0.48  (2.50 to 3.04) | 2.79 ± 0.52  (2.49 to 3.09) | 3.12 ± 0.37  (2.91 to 3.32) | 3.20 ± 0.630  (3.03 to 3.37) | 3.07 ± 0.46  (2.81 to 3.33) |
|  |  |  |  |  |  |  |  |  |  |
| **Log_10_HF** (ms^2^) | TEMP | 2.79 ± 0.49  (2.51 to 3.07) | 1.75 ± 0.74  (1.29 to 2.20) | 1.95 ± 0.53  (1.65 to 2.25) | 2.13 ± 0.54  (1.83 to 2.43) | 2.30 ± 0.60  (1.96 to 2.64) | 2.50 ± 0.50  (2.22 to 2.78) | 2.76 ± 0.54  (2.45 to 3.06) | 2.69 ± 0.55  (2.37 to 3.02) |
|  | HEAT | 2.89 ± 0.43  (2.65 to 3.13) | 1.62 ± 0.69  (1.21 to 2.02) | 1.88 ± 0.53  (1.57 to 2.20) | 2.09 ± 0.54  (1.79 to 2.39) | 2.23 ± 0.41  (2.00 to 2.46) | 2.65 ± 0.50  (2.37 to 2.93) | 2.63 ± 0.45  (2.38 to 2.89) | 2.56 ± 0.61  (2.21 to 2.90) |

Mean ± SD (95%CI). TEMP, temperate condition in which exercise was performed in a room at 22.4°C; Heat, exercise performed in a room at 38.8°C. Log_10_RMSSD, base-ten logarithm of root-mean-square of successive differences of R-R intervals; Log_10_SDNN, base-ten logarithm of the standard deviation of N-N intervals; Log_10_SD1, base-ten of the standard deviation perpendicular to the line of identity; Log_10_SD2, base-ten of the standard deviation parallel to the line of identity; Log_10_LF, base-ten low-frequency power; Log_10_HF, base-ten high-frequency power. N=12 (crossover design)

**Table 3.** Descriptive data related to plasma markers of skeletal muscle microtrauma evaluated in the study.

|  |  | **Baseline** | **30min post-exercise** | **24h post-exercise** | **48h post-exercise** | **72h post-exercise** |
| --- | --- | --- | --- | --- | --- | --- |
| **CK** (U/L) | TEMP | 205.7 ± 86.3  (156.8 to 254.5) | 277.8 ± 114.8  (212.9 to 342.7) | 467.0 ± 268.1  (315.3 to 618.7) | 284.9 ± 161.2  (193.7 to 376.1) | 281.2 ± 182.6  (177.9 to 384.4) |
|  | HEAT | 218.3 ± 125.7  (147.2 to 289.4) | 262.8 ± 181.6  (160.1 to 365.6) | 283.8 ± 164.6  (190.7 to 376.9) | 222.8 ± 112.3  (159.2 to 286.3) | 224.8 ± 120.8  (156.4 to 293.2) |
|  |  |  |  |  |  |  |
| **LDH** (U/L) | TEMP | 540.7 ± 132.1  (466.0 to 615.5) | 630.0 ± 198.8  (517.5 to 742.5) | 656.1 ± 181.2  (553.5 to 758.6) | 508.1 ± 145.9  (425.6 to 590.7) | 580.0 ± 160.5  (489.2 to 670.8) |
|  | HEAT | 502.9 ± 133.4  (427.4 to 578.4) | 585.3 ± 189.7  (477.9 to 692.6) | 453.4 ± 230.5  (322.9 to 583.8) | 419.2 ± 223.3  (292.9 to 545.5) | 570.2 ± 208.5  (452.2 to 688.2) |
|  |  |  |  |  |  |  |

Mean ± SD (95%CI). TEMP, temperate condition in which exercise was performed in a room at 22.4°C; Heat, exercise performed in a room at 38.8°C. CK and LDH, plasma creatine kinase and lactate dehydrogenase concentrations. N=12 (crossover design).
